# Shaping the energy curves of a servomotor-based hexapod robot

**DOI:** 10.1038/s41598-024-62184-y

**Published:** 2024-05-22

**Authors:** Ilya Brodoline, Emilie Sauvageot, Stéphane Viollet, Julien R. Serres

**Affiliations:** 1 Aix Marseille Univ, CNRS, ISM, 163 avenue de Luminy, 13288 Marseille Cedex 09, France; 2 Centrale Marseille, 33 Rue Frédéric Joliot Curie, 13451 Marseille, France; 3 Institut universitaire de France (IUF), 1 Rue Descartes, 75231 Paris Cedex 05, France

**Keywords:** Mechanical engineering, Electrical and electronic engineering, Biomimetic synthesis

## Abstract

The advantageous versatility of hexapod robots is often accompanied by high power consumption, while animals have evolved an energy efficient locomotion. However, there are a lack of methods able to compare and apply animals’ energetic optimizations to robots. In this study, we applied our method to a full servomotor-based hexapod robot to evaluate its energetic performance. Using an existing framework based on the laws of thermodynamics, we estimated four metrics using a dedicated test bench and a simulated robotic leg. We analyzed the characteristics of a single leg to shape the energetic profile of the full robot to a given task. Energy saving is improved by 10% through continuous duty factor adjustment with a 192% increase in power maximization. Moreover, adjusting the robot’s velocity by the step length and associating this with gait switching, reduces the power loss by a further 10% at low-speed locomotion. However, unlike in animals, only one unique optimal operating point has been revealed, which is a disadvantage caused by the low energetic efficiency of servomotor-based hexapods. Thus, these legged robots are severely limited in their capacity to optimally adjust their locomotion to various tasks—a counter-intuitive conclusion for a supposedly versatile robot.

## Introduction

Mobile robots, through their development, are optimized to have the most efficient movements^1–4^ and to increase their range or their ability to walk over more or less complex terrains. In the case of hexapod robots, which are assembled from numerous actuators^5^, power consumption optimization is crucial. Our study aims to demonstrate that there are several ways of optimizing the energetic operating point of a hexapod robot depending on the task or the goal. We are yet to see any simple hexapod robot working side by side with humans. A big technological gap remains between the reality of hexapod robotics and what is expected of them^5^, whereas wheeled autonomous vehicles are currently more energy efficient and can reach greater speeds^6^. On the biological side, there are numerous methods used to estimate the gait performance of humans^7,8^ or animals^9,10^. These methods are, first and foremost, used to understand the laws of living beings’ energy efficiency and locomotion choices^11,12^. Once understood, these laws can be applied to the sport sciences^13^, in order to train an individual to perform a specific task with greater efficiency. However, legged robots can not be trained like athletes, their mechanical designs have to be defined and built for a given task.

Energetic characterization of vehicles has been a topic of controversy since the last century^14,15^. In 1950, G. Gabrielli and Von Karman established the energetic indicator called *specific resistance* $\epsilon$ ($\epsilon = P_{in}/(m\cdot g\cdot v))$^14^, which is defined as the ratio between the power input of the vehicle $P_{in}$ over the product of the weight $m\cdot g$ (*g* is the gravity acceleration) times the speed *v*. More precisely, this ratio comes from the tractive force definition^16^ ($P_{in}/v$) under an efficiency of $100\%$, generally calculated for wheeled vehicles in frictional contact with a surface, such as trains. By dividing the tractive force by the transport weight, we obtain a dimensionless value, the specific resistance $\epsilon$. This value is often inaccurately called Cost of Transport (CoT)^17,18^ and can also be calculated using the output mechanical power of the system^1,19,20^, instead of the input one^21,22^.

The CoT is not a single performance indicator assigned to a vehicle, but a number defined for a specific speed and payload, and it varies depending on the terrain slope^23^ or the chosen gait^24^. Thus, to compare the performance of multiple vehicles, it must be done under similar environmental conditions and loads^5^. Vehicles are also assumed to be similar in terms of class and mass^14,18^, since some transports are designed for low speed and high payload and others for high speed and low payload. In the animal kingdom, a similar value, also named cost of transport ($CoT~[{\text{ml}}/({\text{m}}\,{\text{ kg}})] = \Phi _{{O_{2} }} /({\text{v}} \cdot {\text{m}})$) is often calculated using respiratory measures^18^, which provides information about the oxygen flow $\Phi _{O_2}$ [ml/s] during the activity of an individual.

Recent breakthroughs^25,26^ have suggested a new generic framework based on thermodynamics to provide an energetic description of a system based on a reduced set of energetic descriptors. This framework is able to bridge the differences of definitions between the specific resistance $\epsilon$ and the animal’s CoT. Reconsidering the oxygen flow as proportional to the wasted energy release^25^, we obtain a unique definition of the CoT, a value which is calculable, not only for an animal, but also for a robot, based on its power loss. Using the latter energetic approach, we can investigate the impact of locomotion gait parameters^27^ on the energetic performance of a hexapod robot. By merging simulation results and measurements from our test bench, we can avoid excessive model simplifications encountered in some studies^21^. With our approach, we can now identify which parameters have a higher impact on locomotion efficiency and versatility. More specifically, this study aims to find a way to shape the energetic curves and tune the robot’s energetic profile to a given task. Our method offers, not only a simple process for testing complete robot performance, but also consists of building a four-quadrant plot to visualize this performance, as opposed to the common CoT plot often used in the field of robotics.

The presented method shows that a servomotor-based hexapod robot possesses only a single optimal operating point corresponding to the walk at its maximal speed, a duty factor of 0.5 and using the longest steps possible without inter-limb collision. However, the new method for observing the energetic profile also allows us to optimize the locomotion in the phases under the maximal speed of the robot by fine-tuning the duty factor and the step length and by choosing the gait. The energy loss can be reduced by $10\%$ with a $192\%$ increase in power maximization.

## Results

**Figure 1:**
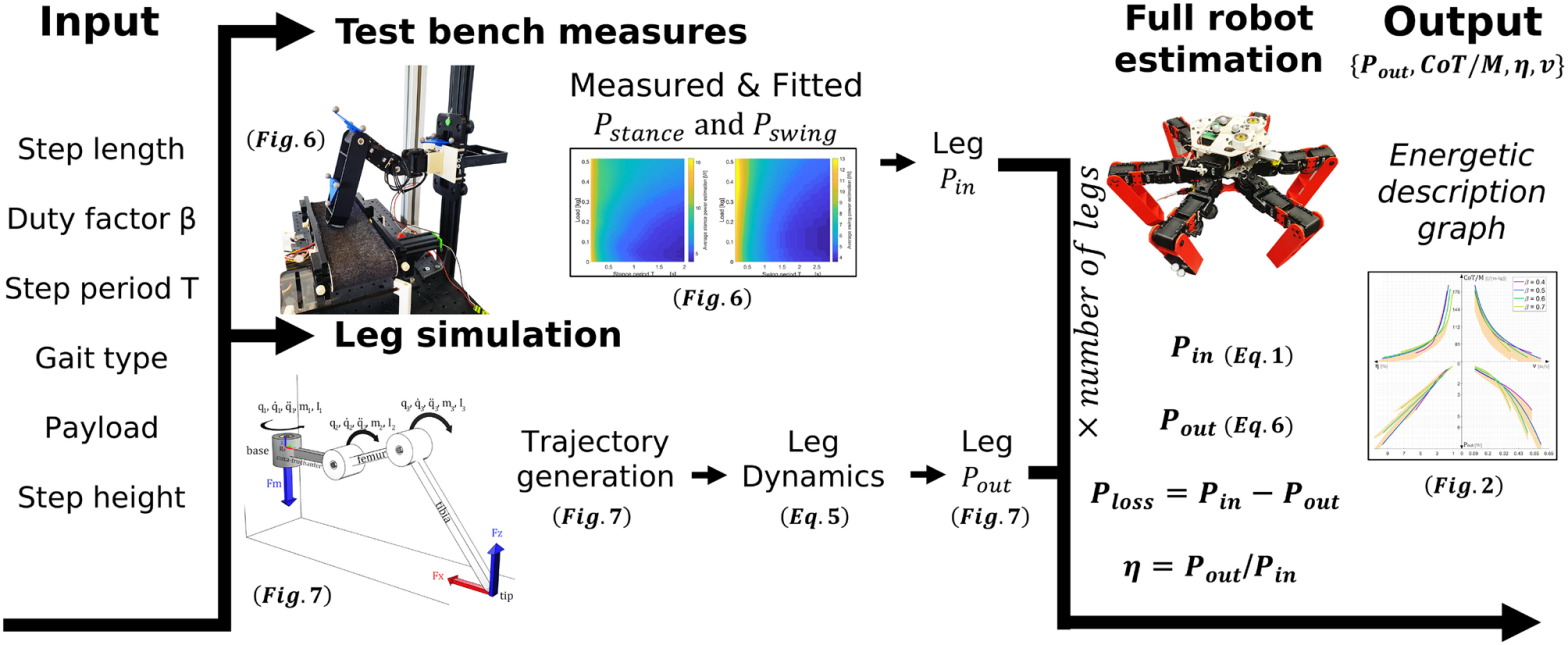
Workflow of the developed method to show the interactions between the various measured and simulated parameters and variables.

In this work, we studied the hexapod robot *AntBot*^28^ with 18 degrees of freedom. Each leg includes three *Dynamixel-AX18A* servomotors. By servomotor, we mean an enclosed compact actuator, composed of a DC motor contiguous to multiple gear stages and including position control circuits. The chosen robot design is a standard case for robots used in studies of animal’s locomotion and navigation^5,28–30^. We focused on relatively small hexapod robots that weigh about 2–3 kg and had a maximum body span of one meter. As shown in Fig. 1, the energetic study of the servomotor-based hexapod robot is done by merging the data collected on our custom built *MiMiC-Ant* test bench^31,32^, merged with single leg numerical simulation data. The test bench consists of a treadmill on which the leg walks and is equipped with three motion capture cameras, a power consumption measurement system, and a thermal camera.. The energy flows of a fully assembled hexapod robot are then estimated under various gait parameters^27^: electrical input power $P_{in}$ (see Eq. 1), mechanical output power $P_{out}$ (Eq. 6), and power loss $P_{loss}$ ($P_{loss}=P_{in}-P_{out}$). In this study, we consider the default gait as tripod, with a 0.5 duty factor $\beta$ and a step length of 100 mm.

### The influence of gait parameters on energy flows

The locomotion of a legged robot is defined by several gait parameters including the duty factor, step length, gait type, and the number of legs. In this work, we investigated the influence of these parameters on the energetic profile of a servomotor-based hexapod robot. Energetic description of the studied hexapod was done through the quantities $\{P_{out}, \eta , CoT/M, v\}$^26^, presented on each graph Fig. 3A–D, where $P_{out}$ is the output mechanical power, generated by the legs’ joints, required for movement and load support; $\eta = P_{out}/P_{in}$ the robot’s efficiency; $CoT=P_{loss}/v$ the cost of transport defined by the power loss and *v* the velocity ($v =$ step length $/(\beta \cdot T)$). In our case, to visualize a value comparable to the specific resistance $\epsilon$, we compute the specific cost of transport *CoT/M* $(CoT/M = P_{loss}/(M\cdot v))$, where *M* is the total robot mass including payload.

First, we examined the influence of the duty factor $\beta$ on the performance of the robot, with a step length fixed at 100 mm. The duty factor is defined as the ratio between the stance phase duration (propulsion) and the leg walk cycle period ($\beta =T_{stance}/T$). Thus, the closer the ratio is to 1, the longer the leg remains in contact with the ground. From this definition, it is clear that a higher $\beta$ value results in more frequent activation of the servomotors, leading to an increase in both the mechanical output power and the robot’s power loss. However, experimental results help us to more precisely understand in which case the change of the duty factor value might be advantageous. As shown in Fig. 3A, the maximum velocities reached by each *CoT*/*M* curve illustrate that, above all, the duty factor has a limiting effect on the walking speed. The duty factor determines the proportions between the stance (propulsion) and swing phases (aerial). Thus, moving away from the middle value (0.5) of $\beta$, the robot’s stance or swing phase duration becomes shorter until it is limited by the actuator’s maximum rotary speed, thereby limiting the walking speed. In the case of our hexapod robot *AntBot*, the minimal swing phase lasts 0.16 s, and the minimal stance phase 0.15 s, which gives, with $\beta$ equal to 0.5, a velocity of 60 cm/s.

Regarding the influence of the duty factor on the energetic profile (Fig. 3A), the main highlighted feature is the increase in efficiency $\eta$ with the increase in speed *v* (limited by $\beta$). The highest efficiency is reached for $\beta$ equal to 0.5. Thus, for a servomotor-based hexapod robot, an increase in speed leads to a larger rise in output mechanical power rather than in power loss. Consequently, this category of robots can be defined as high-speed robots. Especially as, for each value of velocity, there is an optimal duty factor value, for example, at 26 cm/s, a $\beta$ of 0.7 and at 42 cm/s, a $\beta$ of 0.6. This feature is directly linked to the output power curve $P_{out}$, whose maximal value shifts along the velocity axis with the variation of $\beta$. Thus, at low speed, duty factor values $\beta$ greater or less than 0.5 are favorable to energy saving. At high speed, a duty factor close to 0.5 is more appropriate. This behavior is in accordance with similar theoretical research of optimal duty factor in hexapod animals^19,20^. Decreasing the duty factor value with the increasing of the speed, in order to always walk at the local maximal speed seems to be the best strategy to choose in terms of maximizing energy saving and efficiency, particularly when the robot is operating below its maximum speed. On board our robot, switching from a $\beta$ of 0.5 to a $\beta$ of 0.7 at 26 cm/s will provide a reduction in energy loss of around $10\%$ and an increase of mechanical output power of $192\%$.

Secondly, the step length influence was analyzed at a fixed duty factor $\beta$ equal to 0.5. The step length corresponds to the distance the robot travels during the stance period of a leg. An increase in step length is expected to have two effects: an increase in the robot’s speed and an increase in power loss, as the movement of each leg become larger. The specific CoT curve *CoT*/*M* (Fig. 3B) shows that increasing the step length clearly leads to an increase in speed *v*, and thus a higher efficiency $\eta$. Thus, as with the duty factor $\beta$, step length can be adjusted so that the leg trajectory constrains the servomotors to operate closer to their energetically optimal speed. At low speeds (14 cm/s), there is an optimal step length of 100 mm, for which the energy loss is up to $2\%$ lower than a longer step of 140 mm. Additionally, Fig. 3B demonstrates the ability to adjust the maximum mechanical power $P_{out}$ according to the desired speed, this feature can be of interest for the slow transportation of heavy loads. Adjusting the step length appears to have a minimal impact on the energy consumption of a servomotor-based hexapod robot. However, from the point of view of output power maximization, it is best to select a step length for which the actual frequency is maximized, rather than to maintain the length constant.

**Figure 2:**
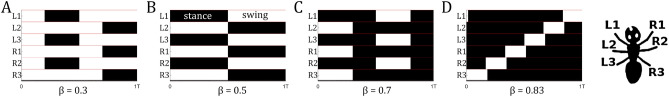
Example of hexapod gait patterns. Legs on the left side of the body are numerated L and those on the right side R. Black stripes correspond to the stance phase (propulsion), white stripes correspond to the swing phase (aerial). (**A**) Tripod gait with a duty factor of 0.3. (**B**) Tripod or wave gait with a duty factor of 0.5. (**C**) Tripod gait with a duty factor of 0.7. **(D**) Wave gait with a duty factor of 0.83.

**Figure 3:**
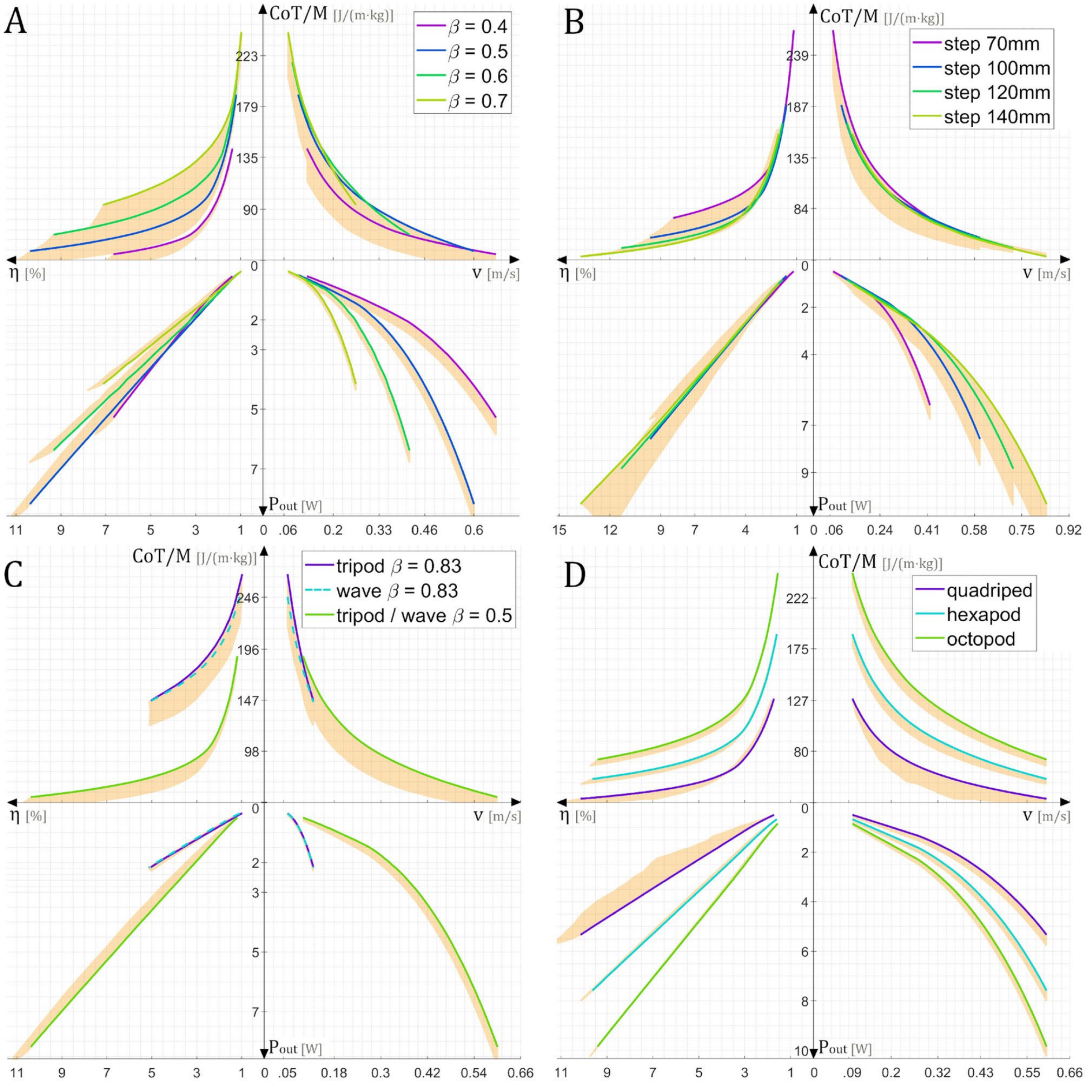
Four-quadrant plot presenting the energetic descriptors $\{P_{out}, \eta , CoT/M, v\}$ of the studied complete hexapod robot. Orange colored area represents the range of variation of the curves when the robot transports loads from 0 to 500 g. As the load increases, each curve slides towards the opposite side of the region. (**A**) Energetic description for various duty factor values $\beta$ when the robot transports no load and walks using the tripod gait. (**B**) Energetic description for various step lengths when the robot transports no load and walks using the tripod gait. (**C**) Energetic description for the tripod gait and the wave gait when the robot transports a load of 140 g with a step length of 100 mm. (**D**) Energetic description of a quadruped, a hexapod and a octopod robot, walking with an alternate gait with a $\beta$ of 0.5, a step length of 100 mm and no load.

Thirdly, we analyzed the influence of the gait type (Fig. 3C). The choice of the gait type determines both the number of legs supporting the body weight at any given instant, and thus the power output. Commonly, a gait is associated to a typical duty factor $\beta$. In our study, we compared the tripod gait with a 0.83 or 0.5 $\beta$ value corresponding to a wave gait (Metachronal rhythm) often implemented on hexapod robots^29,33^ (see Fig. 2). The tripod gait corresponds to the case where the robot has two alternating groups of three synchronized legs. The wave gait corresponds to the walk where the legs move sequentially one after the other. As seen previously, the duty factor is a limiting parameter for the speed, thus the wave gait with a $\beta$ of 0.83 exists only for low speed locomotion, up to 13 cm/s. In this speed range, the energetic loss *CoT*/*M* is up to $8\%$ lower for the wave gait. The wave gait has the characteristic of having only one leg in swing phase, which maximizes the number of supporting legs, an advantage for the transport of heavy loads. Reciprocally, we can say that a gait with a smaller number of ground contacts, such as the tripod or bipod gaits, is more energy-effective for high speed walks.

We also calculated the energy curves for a duty factor $\beta$ of 0.5 (Fig. 3C). As expected, in this condition, both gaits are similar having, at any given moment, three supporting legs and this leads to the same energetic profile. Still, the decrease in CoT for the wave gait could be larger with a different type of actuator designed for high speed movement rather than a high torque output. Estimated data have shown that the input power of the tested robot is only slightly influenced by the transported load. As an example, the average increase in power consumption between a no load walk and a walk loaded by 500 g for the robot walking with a tripod gait with a $\beta$ of 0.5, equals 4 W, nine times smaller than the average power consumption increase from a speed of 10 cm/s to that of 60 cm/s which equals 36 W. This leads to a constant decrease in the specific cost of transport *CoT*/*M* proportional to the increase of load.

Additionally, as our calculations are based on data of a single leg, we compared the performance of our hexapod robot to its quadruped and octopod counterparts (Fig. 3D). As expected, a quadrupedal robot features the lowest specific cost of transport *CoT*/*M*. This is due to the fact that the power consumption of a static robot, i.e., the basal power, contributes significantly to the energy loss. Because the actuators apply a high torque in order to maintain a static position, it is more appropriate to build a quadruped robot than any other variant. However, a higher number of legs provides higher output power $P_{out}$, improving the ability to transport heavy loads and to provide a greater traction.

### The robot’s energetic flexibility

**Figure 4:**
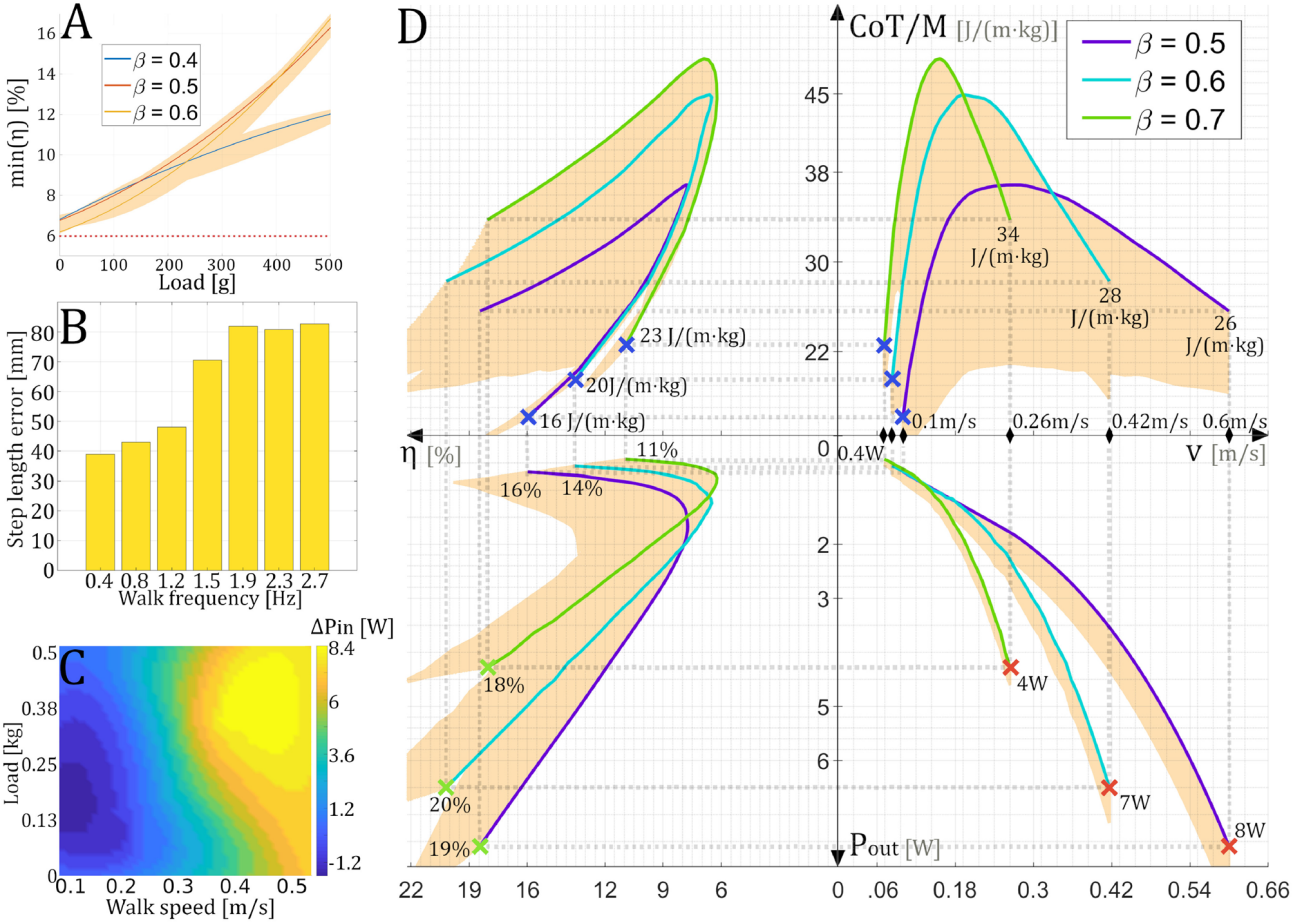
(**A**) Estimated limit of the minimal efficiency $min(\eta )$ which guarantees the existence of multiple optimum operating points for the hexapod robot walking under various duty factors $\beta$. The presented data was collected from a single leg, then extrapolated to the full robot. The orange colored area represents the range of variation of the curves when the robot changes the step length from 70–140 mm. (**B**) Measured average step length error for each walking frequency value from the test bench. Target step length is 100 mm. (**C**) Measured average power variation of the complete robot depending on the walking speed and the transported load after a 10 min walk. (**D**) Four-quadrant plot presenting the energetic descriptors $\{P_{out}, \eta , CoT/M, v\}$ of the hexapod, when the robot possess multiple operating points (based on a hypothetical power reduction of $P_{in}$ by 36 W). The orange colored area represents the range of variation of the curves when the robot transports loads from 0 to 500 g. Red crosses are the maximum power operating points. Blue crosses are the minimum cost of transport points. Green crosses are the maximum efficiency points.

Independently of the gait parameters, the robot achieves its unique optimal operating point (OOP) when it walks at its maximum velocity, as shown in Fig. 3. So in fact, robots are at their most efficient when moving at their maximum speed. In this condition, the robot has the maximum mechanical power output $P_{out}$, maximum efficiency $\eta$, and minimum power loss *CoT*/*M*. By modifying gait parameters, we can adjust the operating point position locally. However, it remains true that for robots only one unique OOP exists, whereas, animals possess multiple OOPs: highest efficiency, highest output power, lowest power loss^26^. Depending on the performed task, an animal will switch to operate at the most suitable working point. Moreover, the same OOP, such as minimum CoT, is reachable for various gait parameters. For instance, horses can switch gait parameters from walking to trotting, whilst keeping the same value of CoT^34^. By and large, having multiple OOPs is an indicator of energetic flexibility, reflecting the ability to overcome environmental challenges. Based on these observations, what conditions would be required to increase our hexapod robot’s energetic flexibility? Our simulations have revealed that a good indicator of the existence of multiple OOPs is the minimal efficiency value $min(\eta )$. When this value exceeds the level of $6\%$, additional optimal operating points emerge. This threshold, corresponds to a minimal condition but not a sufficient one, since the existence of multiple working points depends on the gait parameters such as the step length, the duty factor $\beta$ and the transported load (Fig. 4A).

In a normal state, the robot has a minimal efficiency ($min(\eta )$) between 1.1 and $2.0\%$ (depending on the gait parameters). This extremely low value is due to numerous mechatronical defects, not encountered in the case of living beings. Some of these phenomena, such as actuator backlash, winding and core losses, joint friction, and segment deformations are structural. Others, such as trajectory errors, are caused by inaccuracy in robot’s actuator control. With our *MiMiC-Ant* test bench, we investigated the impact of the main defects shown in Fig. 4B, C, to find out which aspects of the robot should be improved to make it more versatile in performing a variety of tasks.

The efficiency of a robot varies with its mechanical design, the environmental temperature and the duration of walk. As shown by the motion capture data (Fig. 4B), it appears that the step length error increases with the walking frequency. The faster the robot walks, the greater the error. This is due to phenomena not taken into account in the path planning of the leg, such as material resilience, actuator compliance or backlash. We estimated that kinematic errors account for at least $13\%$ of the robot’s total power loss, for a tripod gait with a $\beta$ of 0.5 and a step length of 100 mm. Moreover, the thermal camera recordings presented in Fig. 4C (see the Methods section for details), reveal that the power consumption also changes, during a prolonged walk, with velocity and transported load. The power variation is due to the actuators heating up with use. The graph has to be interpreted as a function of time. The longer the robot walks, the greater the heat and the higher the power consumption, until it reaches the maximum threshold triggering a shutdown or the destruction of a motor. The maximum increase of power per leg is about 1.4 W, which leads to a maximal rise of 8.4 W for a six-legged robot after 10 min. In the case of a robot walking at a $\beta$ of 0.5 and a step length of 100 mm, it represents an average power loss of $6\%$. Besides the kinematic errors and thermal effects, the basal power consumption accounts for an average total power loss of $30\%$. By subtracting the latter from the total robot power consumption, the minimal efficiency ($min(\eta )$) formerly calculated in the range of 1.1–2.0% rises to the range of 1.5–2.6%. Based on Fig. 4A, this range is not sufficient to observe new operating points. This suggests that resolving presented defects isn’t enough to significantly optimize hexapod performance. Consequently, in the case of a robot equipped with small electric servomotors, we can’t have multiple optimal working points. It is not sufficient to optimize the control or even set a resting position, for example by installing springs in the joints. The whole actuator needs to be improved or replaced by a more sophisticated one with higher efficiency.

In the case of an energetically optimized robot, with a minimal efficiency greater than $6\%$, the energetic descriptor curves would be as presented on the Fig. 4D. In our example, this state is reached by reducing the robot’s power consumption by 36 W. Regarding the shape of the curves, we can see two optimal working points for any value of the duty factor $\beta$. One specific velocity corresponds to each point. Selecting the velocity, the robot can operate to maximize the output power $P_{out}$, the cases represented by the red crosses on the graph, such as at 60 cm/*s* for a $\beta$ of 0.5; or minimize the specific cost of transport *CoT*/*M*, the cases represented by the blue crosses on the graph, such as 10 cm/s for a $\beta$ of 0.5. Gait parameters have the same influence as described in the previous section. In addition, the step length and the duty factor also determine if the maximal efficiency is reached for the maximum power or the minimum CoT. A robot, with this energetic profile, could conduct missions at high or low speeds with similar efficiency. This is a behavior expected from hexapod robots, which are often developed for rough terrain exploration.

## Discussion

In this work, we presented an innovative method to predict the performance of a complete servomotor-based hexapod robot based on the analysis of a single leg. We also studied the influence of several locomotion parameters: the duty factor, the step length, the gait type, and the number of legs. The duty factor showed a possibility of reduction in power loss by $10\%$ if it’s continuously adjusted with the speed, thus being favorable to long distance walks. The step length variation was only favorable for reaching a high-speed walking with a slight power loss reduction of $2\%$. However, reducing the step lengths at low speed, would be advantageous to maximize the output power, and thus improve the acceleration or to transport heavier loads. The gait switching showed the advantage of a wave gait in low-speed locomotion under heavy loads, reducing the power loss by $8\%$, and maximizing the mechanical power output. Using the four-quadrant visualization of the energetic profile, we can define the best set of parameters to optimize the hexapod’s walk with respect to local conditions for the transition phases, until it reaches its unique optimal operating point at its maximal speed. For any set of locomotion parameters, the highest speed corresponds to the highest efficiency and power output, and the lowest power loss.

First and foremost, we are going to discuss the precision of the estimations made during this study. Often, the accuracy of the model is not a subject of discussion in research projects grouping simulations with real robot experiments^1,29^. We have chosen a dual estimation method, based on both data provided by simulation and those measured from a real robot leg. Measurements provided by the bench have the advantage of taking into account all the mechatronical defects and non-linearities: actuator backlash, winding and core losses, joint friction, segment deformations, and trajectory errors. On the other hand, simulated robot is only used to estimate the necessary mechanical power, since we control all the environmental and system variables, thus avoiding the defects. The presented method is twice as accurate as a simple simulation using the manufacturer’s specifications of the servomotors (average $P_{in}$ error of $\pm 13~W$), and provides an input power consumption estimation with an average error of $\pm 6~W$. Unlike precise dynamic models^29,35,36^, our method, which is rapidly adaptable to any leg design, offers an easier approach and takes into account kinematic errors and thermal effects over time. These thermal effects also limit power estimation accuracy. As illustrated in Fig. 4C, the average increase of temperature induces an error of $\pm 3.6~W$ after a 10 min walk, and this defines the minimal accuracy of an estimation.

With respect to the results in Fig. 3, our energetic approach helps to choose the optimal gait parameters to shape the energy curves to the task and the environment, and provides more significant indicators to optimize the walk, than just a simple power study^36^ or a specific resistance analysis^1,37^. One of the environmental challenges is uneven terrain navigation, for example on grass, leaves or stones. In this case, our results have shown the wave gait to be more appropriate for slow walk (Fig. 3C), having a greater stability, propulsion and lower energy loss *CoT*/*M* than the tripod gait. However, this observation brings out the issue of definition. A similar robot, built with identical actuators^29^, revealed that the wave gait has a higher specific resistance value $\epsilon$ than the tripod gait, consequently it is less energy efficient. The issue is that the specific resistance is defined using the input power $P_{in}$, which includes the mechanical power $P_{out}$. In the case of the wave gait, which provides greater propulsion $P_{out}$ than the tripod gait, $\epsilon$ value is higher. On the other hand, by using our approach, we can clearly identify the proportion of the input power that is actually used by the robot, and that which is wasted. This overview of the energetic profile seems to be more meaningful. In particular, it can be used to provide additional inputs to current gait transition models^38^ based on terrain roughness or slope detection. The specific resistance and specific CoT have some common properties, such as the fact that both values have a tendency to constantly decrease with the speed increase^1,37^. This phenomenon is due to the actuator’s power consumption, which is dominated by the basal power or first order dynamic effects^37^. Reducing power loss by selecting the highest attainable duty factor $\beta$ for the given speed has also been observed in other hexapod robots with a different leg design^37^. Concerning step length, middle values are usually preferred, since short ones limit the duty factor and speed, and longer ones reduce walk stability^37^.

From a biological point of view, the servomotor-based hexapod has a considerably higher minimal cost of transport value than any animal of similar mass^18,39^. In the category of 2 kg animals, the minimal CoT is constantly under 19 J/(m kg), while our robot reaches the minimal value of 35 J/(m kg) (for *v*: 84 cm/s, no load, step length: 140 mm and $\beta :~0.5$). However, these are still hardly comparable values. Most of the known CoT measures concern quadrupeds, or bipeds. No studies are related to a 2 kg arthropod such as a coconut crab, or to insects with a body span of about 45 cm. Mammals are better at developing energetic optimizations, since their posture allows them to switch between a pendulum type walk and a spring type walk with aerial phases^12,40^. This has inspired the development of various quadruped robots, with advanced power optimization, crossing the 19 J/(m kg) level of cost of transport^41^. Recent ant experiments have, nevertheless, revealed a linearly increasing step length and a decreasing duty factor with the increase in walking speed^42,43^. This behavior accords with our results, showing an optimized power consumption. The existence of multiple operating points has not yet been shown in insects, even though sufficient data exist^10^. However, analogous to fast and slow muscular fibers in animals^44,45^, actuators of varying power categories can be used on a robot to improve efficiency at selected speed ranges. Additionally, new methods are being developed providing a micrometer-scale description of insects’ morphology^46^. Such advances should be beneficial to the development of bio-mimetic designs for legged robots, which will bear their loads on their physical structure rather than carrying them on their actuators.

## Methods

The methodology presented in this study is based on the evaluation of a small servomotor-based hexapod robot, often used for bio-inspired locomotion or navigation purposes^5,28–30^. The hexapod is composed of six identical legs, each of them actuated by three electric *Dynamixel AX-18A* servomotors (with a total of 18 degrees of freedom) with a maximal body span of 45 cm and a mass of 2 kg. The leg segments trochanter-coxa, femur and tibia have the lengths 53 mm, 83 mm and 146 mm, respectively. Our results are based on data collected from three distinct experiments: a study of a complete robot, a session of tests caried out on a single leg with the help of our *MiMiC-Ant* test bench^31,32^, and a numerical simulation of a robotic leg. Each experimental procedure is presented separately in the following sections.

### Complete robot experiment

**Figure 5:**
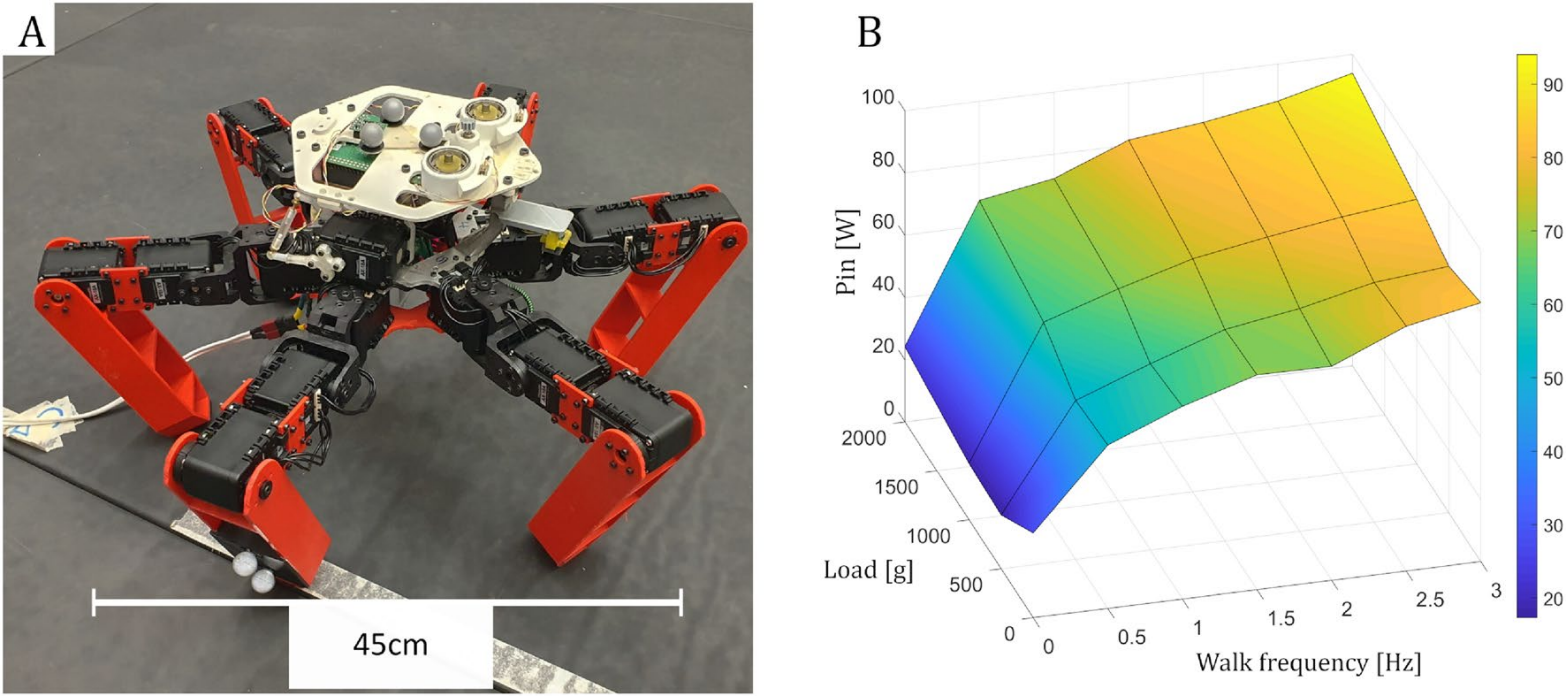
(**A**) AntBot robot in the Mediterranean Flying Arena, equipped with motion capture trackers on the body and one leg. An external power supply substitutes for the battery. (**B**) AntBot robot input power $P_{in}$ measurement results for a step length of 100 mm.

We proceeded to multiple recording sessions of the *AntBot*^28^ hexapod robot (Fig. 5A). The aim was to verify observations and estimations done on the basis of the single leg tests. We then evaluated the accuracy of the method and estimated the effect of step length variation. The robot performed a few sequences of ten steps, on a flat floor, composed of semi-rigid foam mats. During each sequence, one parameter influencing the robot’s walk was changed. First, the walking frequency, from 0 Hz (static robot) to 3 Hz. Frequencies higher than 3 Hz are excluded to avoid inter-legs collisions. Then, the transported load, from 0 to 2 kg (by steps of 500 g), and finally the step length (40 mm, 70 mm, 100 mm, and 120 mm). The step height was kept constant at 40 mm. During each recording sequence, the motions of the body and the middle leg were recorded by a Vicon® motion capture system. Power consumption was recorded by a current clamp meter positioned around the cable supplying the robot’s power. Fig 5B shows an example of collected data.

### Test bench measurements

The complete robot energetic performance estimation is based on a study of a single leg, through our *MiMiC-Ant* test bench^31,32^. The goal of our test bench, is to collect a large amount of data (kinematic, dynamic, energetic), by carrying out several testing sessions on a given robotic leg. All the data are recorded automatically, without human intervention. The advantage of this test bench is to complete numerous recording sessions during a period of several days, which would be a very time-consuming task if done manually on the complete robot. The type of robotic leg installed on the test bench is not specific. The test bench can carry out the tests on any leg, as long as the leg’s communication device is configured according to the requirements and does not exceed the bench’s usable dimensions (for more details, see the Data Availability section).

**Figure 6:**
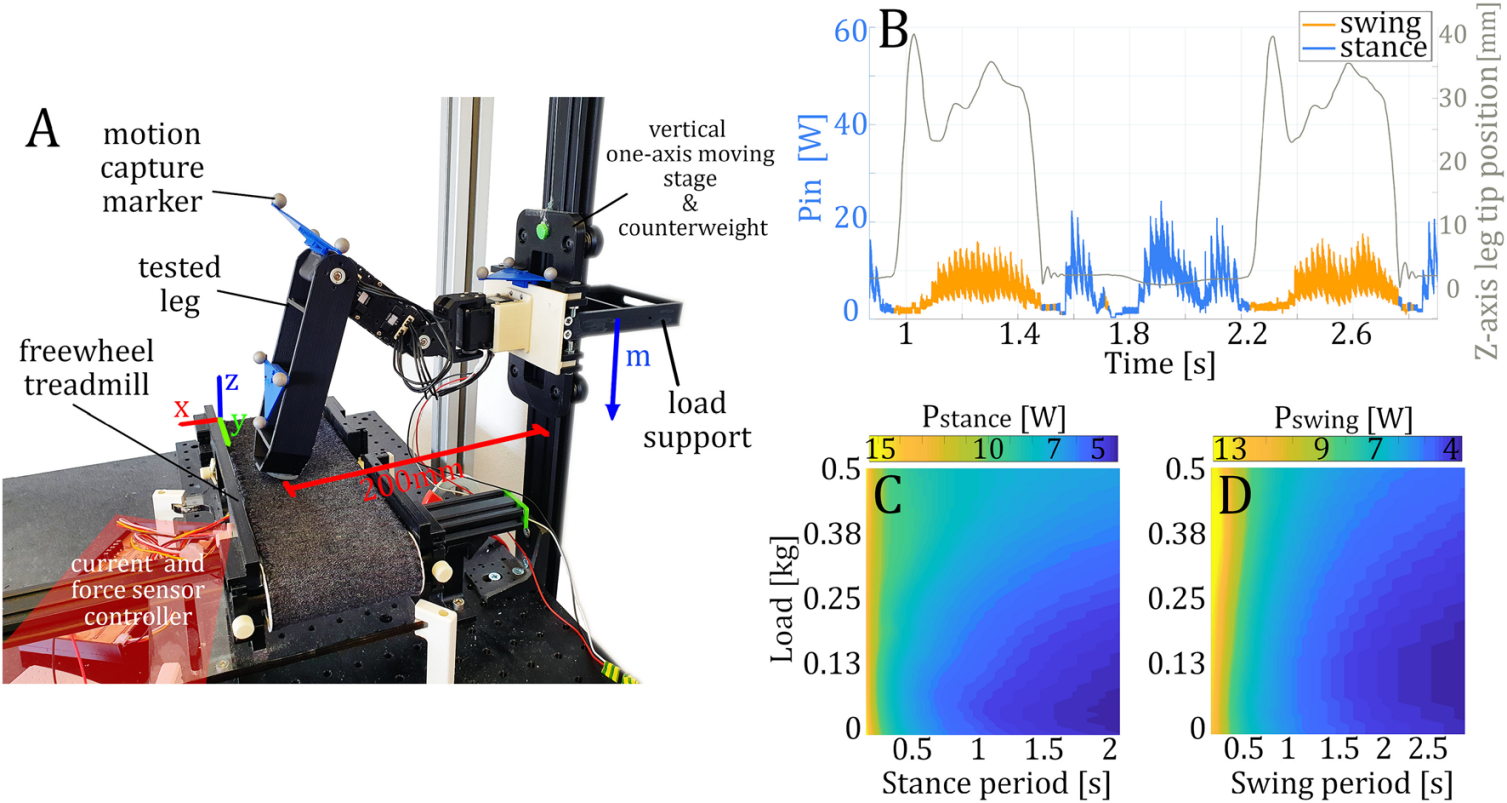
(**A**) Dedicated *MiMiC-Ant* test bench to evaluate energetic consumption of any robot leg. (**B**) Instant power measurement of the leg walking at 0.8 Hz with a load of 100 g and the leg tip position along the vertical Z-axis. The curve is split into swing and stance phase. (**C**) Estimation of the average power consumption of the stance phase $\overline{P_{stance}}(T_{swing},load)$. (**D**) Estimation of the average power consumption of the swing phase $\overline{P_{swing}}(T_{swing},load)$.

The *MiMiC-Ant* test bench is presented in Fig. 6A. The structure of the bench was inspired by study procedures done on quadruped animals^11^ and insects^9,10^. The test bench is composed of a set of three Miqus M3 Qualisys™ motion capture cameras, a normal ground force sensor embedded inside a horizontal freewheel treadmill, a vertical moving stage, and a point of attachment for the leg. In this way, the test bench is able to record kinematic data describing the movement of the walking leg on the treadmill along with ground reaction forces under various loads attached to the vertical moving stage. Additionally, the test bench keeps track of the power supply current and voltage. The whole setup is in a temperature controlled environment, supervised by a thermal camera FLIR Lepton 2.5, which tracks the average and maximum temperatures. This camera also measures heat increases in the tested leg to identify the thermal issues of the leg design.

The experimental procedure used to collect the data includes multiple sessions of 10-minute walks. Each session corresponds to a different value of transported load. The experiment, managed by the main computer, is composed of the following steps:

1. First, a load is attached to the specially designed support on vertical moving stage. In our experiment, we used five loads: 0, 95 *g*, 141 *g*, 187 *g* and 515 *g*. A load of zero, corresponds to the case when the leg support only its own mass. The chosen values represent the limits of the estimation model.
2. Once a load is present, it’s necessary to manually position the stop mechanism, which sets the lowest vertical position of the leg. The stop prevents the leg from falling to the floor during the aerial phases. Its position depends on the leg design, since the overall leg structure can bend differently under load.
3. Environmental and robot leg temperature are checked. Recording starts only if the system-defined temperature value is reached, in our case $26~^\circ C~\pm 1~^\circ C$. This security guarantees that each experimental session starts under the same initial conditions, since the temperature of the motors has an influence on energy consumption (Fig. 4C) and limits the time of use due to overheating.
4. The leg starts to walk on the freewheel treadmill at one of the defined frequency. In our experiment, we used eight different frequencies, from 0 to 3 Hz. A session is run for the frequency of 0 Hz, i.e. static robot, in order to measure the basal residual energy consumption^26^. From the point of view of motion control, the leg is performing exactly the same movements as on the full robot, with a step length fixed at 100 mm. The duty factor has no importance, since the stance and swing phases are split afterwards. In our case, the duty factor was set to 0.5. During the walk, the leg isn’t locked vertically, which leads to vertical oscillations similar to those experienced on a complete robot body. Thus, walk characteristics estimated through these measurements correctly match the real characteristics of the robot.
5. After a 10 min walk, the leg is stopped and a cool-down is initiated by switching off the power supply. After approximately 30 min, the initial temperature of $26~^\circ C~\pm 1^\circ C$ is reached. Deactivation of the power supply is important in the case of servomotor-based robots because, with this type of actuation, the leg still consumes power, even in a static position, creating a constant temperature increase until the servomotor overheats.
6. Previous steps are repeated until the end of all the desired sessions for each speed and load combination.

Collected data, saved in a database, are processed as shown in Fig. 6. Data are split by separating the input power of the leg into two groups, swing power and stance power, by means of the force sensor and tracking markers positions. The first group corresponds to the aerial leg movement and the second one to the propulsion movement (Fig. 6B). Then, using the recorded data for each speed and load, we are able to do a surface fitting (Fig. 6C-D). The fit predicts the average power consumption of each phase $\overline{P_{stance}}(T_{stance},load)$ and $\overline{P_{swing}}(T_{swing},load)$, for any desired gait defined by a load, a speed and a duty factor. Since we fit the power values depending on the phase time and not the robot speed, we are able to build combinations of stance and swing periods to compute any desired duty factor.

We can then calculate, from single leg average power estimations, the total average input electric power $P_{in}$ of the fully assembled robot through Eq. 1.

1 $$\begin{aligned} \overline{P_{in,T}} = \frac{6}{T} \cdot \left( \int _{0}^{\beta \cdot T} \overline{P_{stance}}(\beta \cdot T,m_{base}(t)) \cdot dt + \int _{\beta \cdot T}^{T} \overline{P_{swing}}((1-\beta ) \cdot T,m_{base}(t))\cdot dt\right) \end{aligned}$$

with $T = T_{stance} + T_{swing}$ the walk period, $T_{stance}$ and $T_{swing}$ the periods of the stance and swing phases, respectively, and $\beta = T_{stance}/T$ the duty factor. The value $m_{base}(t)$ defines the mass carried by each leg (Eq. 2).

2 $$\begin{aligned} m_{base}(t) = \frac{m_{load} + (6-L(t))\cdot m_{leg}}{L(t)},\qquad \qquad t\in [0,T] \end{aligned}$$

with *L*(*t*) a function giving the number of supporting legs at each instant *t* (Eq. 3). This function depends on the period *T*, the duty factor $\beta$, and the legs relative phases $\theta _{i} \in [0,1]$ ($i\in [1,6]$), which is a number associated to each of the 6 legs, defining the starting time of the stance phase with respect to the beginning of the walk period.

3 $$\begin{aligned} L(t) = \sum _{i=1}^{6}\left( 1-\left( sgn\left( sin\Bigg (\frac{(t - \theta _i\cdot T))\cdot \pi }{T}\right) \cdot sgn\left( sin\Bigg (\frac{(t - (\theta _i + \beta )\cdot T))\cdot \pi }{T}\right) +1\right) /2\right) \end{aligned}$$

*L*(*t*) can be calculated in various ways, but Eq. 3 defines *L*(*t*) simply as a sum of six rectangular waveforms, one per leg, with a duty cycle proportional to the duty factor $\beta$, temporally offset by the relative phase $\theta _i$. In the case of the commonly used tripod gait, with $\beta$ equal to 0.5, we have $L(t) = 3$, $\forall t\in [0,T]$.

### Simulation

**Figure 7:**
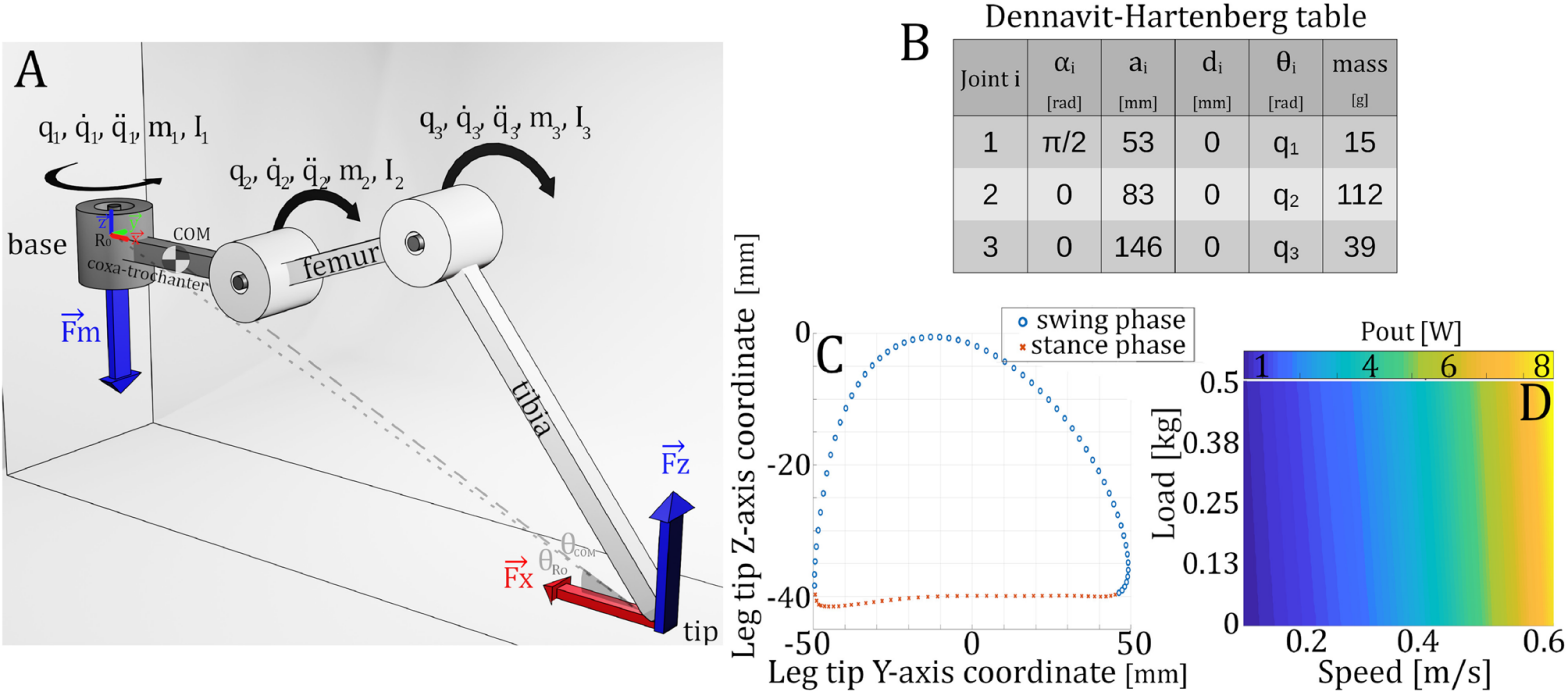
(**A**) Model of the simulated leg made with 3 degrees of freedom. $\vec {Fx}$ and $\vec {Fz}$ are the ground reaction forces. $\vec {Fm}$ represents the force engendered by the load. (**B**) Dennavit-Hertenberg table defining the kinematic chain of the simulated leg. (**C**) Implemented leg tip trajectory used for inverse kinematics calculations. (**D**) Simulated average power consumption of the hexapod robot.

In our study, a simulated robotic leg was used to compute the output mechanical power $P_{out}$ of the walking hexapod robot. More precisely, it represents the power which is necessary to make the robot move and support the load. By definition, we take the leg movement into account, and this is not considered as a flaw, since it is essential to the walk. A simplified model of the leg, composed of three rotary joints representing each servomotor, was built on Matlab® using the Peter Corke *Robotics Toolbox*^47^. Because most commercial servomotors do not come with a highly detailed specifications sheet, we were unable to simulate friction, gear ratio, motor axis inertia, inductance and resistance in our modeled motor. We simulated the rotary joints as proxies, corresponding to the case of ideal motors. Fig 7A represents the leg model used in the simulation, and we can see the Dennavit-Hertenberg table defining the kinematic chain of the simulated leg in Fig. 7B. The trajectory, defined in Fig. 7C, corresponds to the one used on the real robot. The path is calculated using the cubic spline interpolation method to guarantee continuity of velocities and accelerations. The leg tip trajectory is split into two sections, corresponding to the swing and the stance phases. Each section is used separately to simulate two dynamic behaviors since it is only during the stance phase that the ground reaction forces (Eq. 4) act on the tip of the leg.

4 $$\begin{aligned} \vec {Fz} = (m_{leg}+m_{load}) \cdot g \cdot \vec {z} \qquad \qquad \vec {Fx} = -(m_{leg} + m_{load})\cdot g\cdot \frac{cos(\theta _{COM})}{sin(\theta _{R_0})}\cdot \vec {x} \end{aligned}$$

where $m_{leg}$ and $m_{load}$ are the known masses of the leg segments and of the additional mass, *g* is the gravitational acceleration constant, $\theta _{COM}$ is the angle defined by the $\vec {x}$-axis, the leg tip point and the center of mass (COM) estimated at each leg position. The cosine of this angle corresponds to the vertical distance between the leg tip and the COM, $\theta _{R_0}$ is the angle defined by the $\vec {x}$-axis, the leg tip point and the base frame origin of the leg. The sine of this angle corresponds to the horizontal distance between the leg tip and the origin base frame $R_0$.

The third ground reaction force component $\vec {Fy}$ is neglected, as the robot is considered to walk at a constant speed and as we ignore the starting and stopping phases of the walk. With these hypotheses, the body’s inertia doesn’t affect walk dynamics in the direction parallel to the walk. This phenomenon is represented on the test bench (Fig. 6A) by the presence of a freewheel treadmill, which has a high friction tread made of fabric and moves with the silicon leg tip without sliding (measured static friction $\mu _{s}=$ 0.8). The tread is stretched between two bearings, and rotates with negligible friction.

Once the simulation model is defined (see details in Supplementary Information), the Lagrange-Euler Formulation^47^ is used to estimate the average mechanical power output of the robot leg during one walking period *T* (Eq. 6).

5 $$\begin{aligned} \left[ {\begin{array}{c} \tau _{1}(t)\\ \tau _{2}(t)\\ \tau _{3}(t)\\ \end{array} } \right]= & {} M(q(t)) \ddot{q(t)} + C(q(t),\dot{q(t)})\dot{q(t)} + G(q(t)) + J(q(t))^{T}f(t)\qquad \qquad t\in [0,T] \end{aligned}$$

6 $$\begin{aligned} \overline{P_{out,T}}= & {} \frac{6}{T} \cdot \int _{0}^{T} \sum _{i=1}^{3} \mid \tau _{i}(t) \cdot \dot{q_{i}}(t)\mid \cdot dt \end{aligned}$$

  where $\tau _{i}$ are the resulting torque values of each joint, $q(3\times 1),~{\dot{q}}(3\times 1),~\ddot{q} (3\times 1)$ are respectively the angular position, velocity and acceleration of each leg joint, $M(3\times 3)$ is the joint-space inertia matrix of the robot leg, $C (3 \times 3)$ is the Coriolis and centrifugal terms matrix, $G (3 \times 1)$ is the gravity terms matrix, $J (6 \times 3)$ is the leg Jacobian, $f (6\times 1)$ is the wrench vector composed of forces and moments applied at the leg tip (ground reaction forces), *g* is the gravitational acceleration constant, and *T* is the walk period of the robot.

Using the previously defined leg model, the full robot output power was computed for walking speeds varying from 0.1 to 0.6 m/s and loads from 0 to 500 g. The step height was kept constant at 40 mm. The resulting power map is presented in Fig. 7D.

## Supplementary Information

Supplementary Information.

## Data Availability

Additional technical details are available on our GitHub Repository: https://github.com/IlyaBrod/MiMiC-ANT-testbench. The datasets used during the current study are available from the corresponding author on reasonable request.
