# Supplementary material for: Shaping the energy curves of a servomotor-based hexapod robot
Source: Sci Rep. 2024 May 22;14:11675. doi: 10.1038/s41598-024-62184-y (PMC11636921; doi:10.1038/s41598-024-62184-y)
Supplement: Supplementary file 1 — Supplementary Information. [file 41598_2024_62184_MOESM1_ESM.pdf]

Shaping energy curves of a servomotor-based hexapod robot

Ilya Brodoline[1,*], Emilie Sauvageot[1,2], Stéphane Viollet[1], and Julien R. Serres[1,3]

[1] Aix Marseille Univ., CNRS, ISM, Marseille, France
[2] Centrale Marseille, 33 rue Frédéric Joliot Curie 13451 Marseille, France
[3] Institut universitaire de France (IUF), 1 rue Descartes, 75231 Paris cedex 05, France
* ilya.brodoline@univ-amu.fr , ilya.brodoline@gmail.com

Supplementary Information

Robot leg model

Supplementary Table S1. Leg model parameters

Joint i	Joint type	Length [mm]	Mass [g]	Center of mass O_iG_i [m]	Inertia tensor with respect to the center of mass [kg·m²]
0 – body (no payload)	-	-	148	$\begin{bmatrix}0\\0\\0\end{bmatrix}$	-
1 – coxa-trochanter	Rotary	53	15	$\begin{bmatrix}-0.0265\\0\\0\end{bmatrix}$	$\begin{bmatrix}5.8935e-06 & 3.2000e-10 & 0\\3.2000e-10 & 5.0617e-05 & 0\\0 & 0 & 4.6509e-05\end{bmatrix}$
2 – femur	Rotary	83	112	$\begin{bmatrix}-0.0406\\0\\-0.0010\end{bmatrix}$	$\begin{bmatrix}3.2764e-05 & 1.6000e-09 & -1.8525e-05\\1.6000e-09 & 8.4231e-04 & 2.7390e-07\\-1.8525e-05 & 2.7390e-07 & 8.5125e-04\end{bmatrix}$
3 – tibia	Rotary	146	39	$\begin{bmatrix}-0.0747\\0\\0.0041\end{bmatrix}$	$\begin{bmatrix}1.6836e-05 & 1.4000e-10 & 0\\1.4000e-10 & 9.5062e-05 & 0\\0 & 0 & 8.4081e-05\end{bmatrix}$

The payload values are 0, 95g, 141g, 187g and 515 g + 380g (sensors embedded on the robot).

Leg trajectory parameters

The trajectory points used for cubic interpolation are:

- Leg tip vertical position:
  Normalized timings: `[0 0.1 0.3 0.5 0.85 1]`
  Normalized coordinate: `[1 1 1 0 0 1]`

- Leg tip longitudinal position:
  Normalized timings: `[0 0.1 0.3 0.5 0.85 1.0]`
  Normalized coordinate: `[-0.5 -0.5 0.5 0.5 -0.5 -0.5]`
  (0.5 is the position of the center)

Once the trajectory is interpolated, the coordinates are scaled by the step parameters:

Step height: 40mm
Step distance form body: 180mm
Step length: 70mm, 100mm, 120mm, and 140mm

Gait legs support example

An example of the L(t) function is shown in Fig.S2, in the case of a wave gait with a duty factor $\beta = 0.3$.

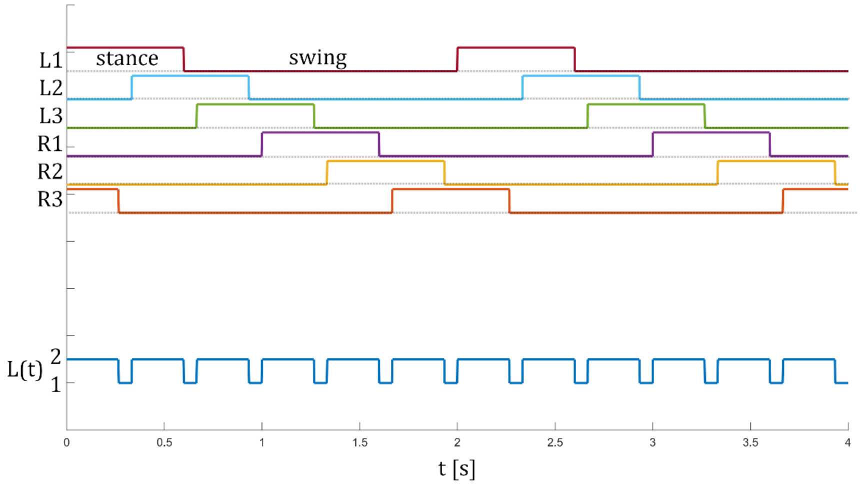

Supplementary Figure S1. Hexapod robot legs state and resulting function L(t) giving the number of legs in stance phase.

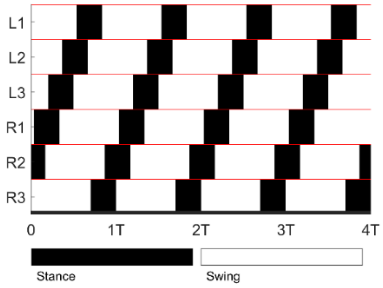

Supplementary Figure S2. Wave gait pattern (duty factor $\beta = 0.3$)

Ground reaction forces calculations

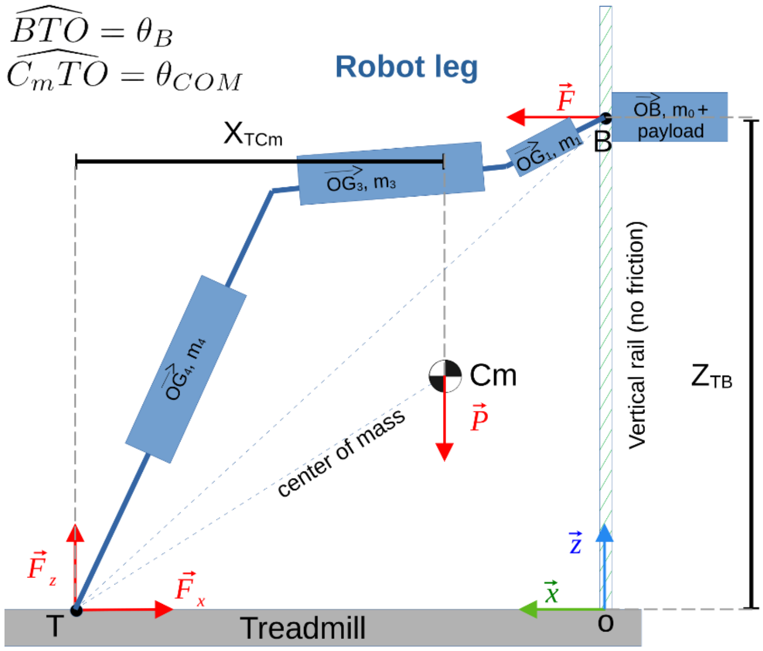

Supplementary Figure S3. Scheme representing the robot's leg in the test bench.

The center of mass is calculated at each leg position, based on the mass values Tab. S1:

$$\frac{1}{\sum_{i=0}^{i=3} m_i} \cdot \sum_{i=0}^{i=3} m_i \cdot {}^{0}T_i \cdot (\mathrm{O_iG}_i)$$

With ${}^{0}T_i$ transformation vector from the reference frame *i* to the reference frame $(B, \vec{x}, \vec{y}, \vec{z}\,)$.
Inertia effects are included in the Matlab simulation, we don't take account of them.
Writing the equilibrium equations in the no slip case, we have:

$$\text{Linear momentum balance } \Sigma\vec{F} = \vec{0}$$

$$\leftrightarrow \begin{cases} on\ \vec{x}:\ F = F_x \\ on\ \vec{z}:\ F_z = P = (m_{leg} + m_{load}) \cdot g \end{cases}$$

$$\text{Angular momentum balance } \Sigma\vec{M} = \vec{0}$$

$$\overrightarrow{TC_m} \wedge \vec{P} + \overrightarrow{TB} \wedge \vec{F} = \vec{0}$$

After projection on $\vec{y}$, we get: $X_{TC_m} \cdot P = Z_{TB} \cdot F_x$

This equation is reformulated as a function of the angles: $F_x = P \cdot \frac{\cos(\theta_{COM})}{\sin(\theta_B)}$

Note: In this article (Fig.6A) the $\vec{x}$ axis is in the opposite direction compared to calculations presented in Fig.S4.
Then, the force vector $[F_x \quad 0 \quad F_z]$ defined in the global reference frame $(O, \vec{x}, \vec{y}, \vec{z}\,)$ is written in the robot's end effector reference frame. We use the transformation matrix ${}^{3}R_1$ (Tab. S1), provided by the *Matlab* toolbox: $F_{endEffector} = {}^{3}R_1 \cdot [F_x \quad 0 \quad F_z]^{\,T}$
